# Preliminary Assessment of Red Beetroot Supplementation and Cultivar Effects in Low-Protein-Fed WKY Rats

**DOI:** 10.3390/nu18122016

**Published:** 2026-06-21

**Authors:** Michał S. Majewski, Anetta Hanć, Magdalena Krajewska-Włodarczyk, Joanna Majkowska-Gadomska, Anna Francke

**Affiliations:** 1 Department of Pharmacology and Toxicology, Faculty of Medicine, University of Warmia and Mazury, 10-082 Olsztyn, Poland; 2 Faculty of Chemistry, Department of Trace Analysis, Adam Mickiewicz University, 61-614 Poznań, Poland; 3 Department of Rheumatology, School of Medicine, Collegium Medicum, University of Warmia and Mazury, 10-082 Olsztyn, Poland; 4 Faculty of Agriculture and Forestry, Department of Agroecosystems and Horticulture, University of Warmia and Mazury, 10-718 Olsztyn, Polandafrancke@uwm.edu.pl (A.F.)

**Keywords:** body composition, body weight gain, cardiovascular function, feed efficiency, Langendorff heart assay, lean body mass, low–protein diet, selenium foliar application

## Abstract

**Background/Objectives**: Red beetroot (*Beta vulgaris* L.) is recognized for its antioxidant, anti-inflammatory, and metabolic properties. This study evaluated the effects of two beetroot cultivars (*Boldor* and *Wodan*) on blood serum parameters, body composition, and organ weights in male WKY rats fed a low-protein diet (LPD, 8.8% protein). **Methods**: Five-week-old male rats were maintained on an LPD for 8 weeks and subsequently continued on the LPD diet supplemented with 4% dried beetroot for 45 days. The experimental diets included beetroot from the *Boldor* and *Wodan* cultivars, either treated or untreated with a plant growth stimulator during cultivation. **Results**: Foliar application of the selenium-based plant growth stimulator did not significantly increase selenium or other element concentrations in beet roots. Elemental analysis showed higher levels of Fe, Zn, Cu, Cr, Pb, As, Cd, and Sb in the *Wodan* group, while *Boldor* increased Cr, Pb, and As; Ni and Se remained unchanged. Beetroot supplementation significantly affected 14 of the 30 measured biochemical parameters, including biomarkers of liver function (ALT, ALP, total bilirubin, albumin, and total protein), renal function (uric acid), pancreatic activity (amylase and lipase), electrolyte balance (sodium, potassium, and chloride), mineral metabolism (calcium), inflammatory status (CRP), and nutritional metabolism (iron). Conversely, no significant effects were observed on lipid profile parameters or biomarkers of cardiac and skeletal muscle injury. Among the beetroot cultivars evaluated, *Wodan* exerted distinct effects relative to *Boldor*, resulting in higher circulating total bilirubin and potassium concentrations, alongside reduced uric acid and lipase levels in treated rats. *Boldor* supplementation significantly increased body weight gain and fat mass, with a trend toward higher lean mass, and increased kidney weight. *Wodan* did not significantly affect body weight but increased kidney and spleen mass. Feed intake was similar across groups. No changes in cardiovascular function were observed ex vivo. **Conclusions**: Beetroot supplementation modulated multiple metabolic and physiological biomarkers in rats fed a low-protein diet, with distinct cultivar-specific effects, underscoring the importance of cultivar selection for optimizing functional dietary interventions.

## 1. Introduction

Beetroot (*Beta vulgaris* L.) is widely consumed as a vegetable and has attracted growing scientific interest due to its rich content of biologically active compounds [1]. Beet root contains a diverse array of nutrients and phytochemicals, including dietary nitrates, betalains, polyphenols, vitamins, and trace elements [1,2,3,4], all of which have been associated with various beneficial physiological effects [1,2]. These constituents exhibit antioxidant, anti-inflammatory, and metabolic regulatory properties and may modulate cardiovascular, metabolic, and immune functions [1,2]. Consequently, beetroot and its derivatives have been increasingly explored as functional food ingredients and dietary supplements [1,2].

Among the bioactive compounds present in beetroot, inorganic nitrate has been extensively investigated [4,5]. Once ingested, nitrate can be converted into nitric oxide (NO), a key signaling molecule involved in the regulation of vascular tone [6], mitochondrial efficiency, and cellular metabolism [5]. Dietary nitrate supplementation has been shown to improve endothelial function, lower blood pressure, and enhance exercise performance in both animal and human studies [4,5]. In addition to nitrates, betalains—water-soluble pigments responsible for the characteristic red coloration of beetroot—exhibit strong antioxidant activity and may play a role in mitigating oxidative stress and inflammatory processes [1,2].

The biological effects of beetroot may also depend on its mineral composition, especially K, Ca, Fe, Mn, and Zn. Trace elements present in plant tissues contribute to metabolic regulation and antioxidant defense mechanisms. Selenium, in particular, is an essential micronutrient incorporated into selenoproteins that are involved in redox homeostasis, immune function, and thyroid hormone metabolism. Selenium deficiency has been linked to impaired antioxidant capacity and increased susceptibility to metabolic disorders. Therefore, agronomic biofortification strategies aimed at enhancing selenium content in plant-based foods have gained considerable attention as a means of improving crop nutritional quality. However, selenium toxicity has also been reported [7].

The chemical composition of beetroot, including its trace element profile, can vary significantly depending on cultivar, environmental conditions, and agricultural practices. Different cultivars of beetroot (e.g., *Boldor*, *Wodan*, *Anello*, and *Avalanche*) may accumulate distinct mineral and phytochemical profiles, potentially influencing their biological activity when used as dietary supplements. Despite increasing interest in beetroot as a functional food, comparative studies evaluating the physiological effects of different cultivars, particularly in the context of selenium foliar application, remain limited [8,9,10,11,12].

Nutritional status and dietary composition can substantially influence physiological responses to dietary interventions. A protein-restricted diet was employed as a nutritional challenge model because inadequate protein intake disrupts nitrogen metabolism, impairs nutrient utilization efficiency, alters body composition, and compromises metabolic homeostasis. Protein deficiency has also been associated with increased oxidative stress, impaired immune function, and disturbances in gut microbial composition, all of which may adversely affect growth and health [13,14,15,16,17]. Plant-derived bioactive compounds, including polyphenols, flavonoids, and other phytochemicals, have been reported to modulate antioxidant defenses, inflammatory responses, and metabolic pathways involved in nutrient utilization. Therefore, evaluating these compounds under conditions of protein restriction may provide valuable insight into their capacity to enhance adaptive responses to nutritional stress, improve nutrient-use efficiency, and mitigate the adverse physiological consequences of suboptimal protein intake. Investigating the interaction between plant-derived supplements and low-protein diets may thus contribute to the development of nutritional strategies that promote metabolic resilience and sustain physiological performance under protein-limiting conditions.

The study aimed to evaluate the potential of beetroot supplementation to modulate metabolic and cardiovascular parameters under conditions of dietary protein restriction.

## 2. Materials and Methods

### 2.1. Drugs and Chemicals

Acetylcholine chloride (CAS No. 60–31–1) and noradrenaline hydrochloride (CAS No. 329–56–6) were purchased from Sigma–Aldrich (St. Louis, MO, USA).

Stock solutions (10 mM) were prepared in distilled water, except for noradrenaline, which was dissolved in a NaCl and ascorbic acid solution (0.9% and 0.01% *w*/*v*, respectively). The final solvent concentration did not exceed 0.01% (*v*/*v*).

All stock solutions were stored at −20 °C. On the day of the experiment, working solutions were prepared using Krebs–Henseleit buffer containing (in mM): NaCl 115, CaCl_2_ 2.5, KCl 4.6, KH_2_PO_4_ 1.2, MgSO_4_ 1.2, NaHCO_3_ 25, and glucose 11.1.

### 2.2. Experimental Design

The study was conducted as a two-factor experiment.

The first factor consisted of two cultivars of beetroot:

- *Boldor*—yellow–orange roots, high uniformity;
- *Wodan*—red roots.

The second factor was foliar application of the selenium-based plant growth stimulator (AminoSelenit):

- Control (1)—water spray only;
- AminoSelenit treatment (3)—3.0 L/ha in 300 L water solution.

Experimental Groups:

*Boldor* 1 (L)—*Boldor* without AminoSelenit;

*Boldor* 3 (M)—*Boldor* with AminoSelenit;

*Wodan* 1 (P)—*Wodan* without AminoSelenit;

*Wodan* 3 (R)—*Wodan* with AminoSelenit.

### 2.3. Materials

#### 2.3.1. Plant Growth Stimulator

The plant growth stimulator used in this study was AminoSelenit, a selenium-containing formulation enriched with plant-derived amino acids. The product is designed to enhance plant tolerance to environmental stress, stimulate metabolic processes, and promote growth and development.

AminoSelenit was supplied as a liquid suspension by Arkop (Bukowno, Poland). The formulation contains 1.4 g/kg selenium (0.14%), 55 g/kg total amino acids, 9.3 g/kg nitrogen, and 50 g/kg organic carbon, with a pH range of 4.0–5.0.

For field application, the product was diluted to a total spray volume of 300 L/ha (2.5 L/ha AminoSelenit + 297.5 L/ha water).

#### 2.3.2. Field Experiment

The field experiment was conducted in 2024 at the Didactic and Experimental Station of the University of Warmia and Mazury in Olsztyn, Poland. The soil at the experimental site was classified as brown soil of quality class IIIb.

Certified seeds of beetroot were obtained from Bejo Zaden (Olsztyn, Poland).

Throughout the growing season, crop management followed standard agronomic practices recommended for beetroot cultivation. To account for spatial variability within the field, the experiment was arranged in a randomized complete block design with four replicates per treatment. Each experimental plot covered an area of 10 m^2^ and was separated by buffer zones to minimize spray drift between adjacent treatments. Foliar applications of the selenium-based plant growth stimulator were performed using a fine mist sprayer under favorable weather conditions (low wind speed), typically during early morning or late evening hours, to enhance spray retention and absorption by plant tissues. The crop was managed in accordance with the principles of integrated table beet production. Foliar treatments were applied three times during the growing season at BBCH growth stages 31, 37, and 42, according to the BBCH phenological scale. No visible symptoms of plant diseases or pest infestations were observed during the experimental period. Weed control was carried out manually throughout the growing season. Beet roots were harvested at physiological maturity during the first ten days of October. Harvesting was carried out when the roots reached an average diameter of 5–8 cm and leaf senescence became visible. From each experimental plot, 8 kg of representative roots were collected. The harvested roots were thoroughly washed to remove soil residues and then dried at 65 °C until constant weight using a Binder ED400 drying oven (Binder GmbH, Tuttlingen, Germany). The dried plant material was subsequently ground into a fine powder using a Grindomix GM300 knife mill (Retsch GmbH, Haan, Germany). The prepared samples were used for further chemical analyses.

#### 2.3.3. Animals

Male Wistar–Kyoto rats (WKY/KyoRj), aged 5 weeks, were obtained from Janvier Laboratories (Le Genest-Saint-Isle, France). Upon arrival, animals underwent routine clinical assessment to confirm good health status and absence of visible abnormalities. Rats were then randomly allocated to five experimental groups (n = 10 per group). Animals were housed individually in stainless steel cages under controlled environmental conditions (22 ± 1 °C, 60 ± 5% relative humidity, 12 h light/dark cycle, ~15 air exchanges per hour). Environmental enrichment (nesting material, wooden chew sticks, and toys) was provided to promote natural behavior. Food and water were available ad libitum throughout the study.

The sample size was determined based on a priori statistical power analysis for a one-way ANOVA design with five experimental groups. Assuming a significance level of α = 0.05, a statistical power of 80% (1 − β = 0.80), and a large expected effect size, the minimum required sample size was estimated to be 10 animals per group. Therefore, a total of 50 animals were included in the study.

Animals were monitored daily for general health status, behavior, and body weight. Humane endpoints were predefined, and animals exhibiting severe distress, illness, or body weight loss exceeding 15% were euthanized. All procedures were conducted by trained personnel in accordance with established guidelines for the care and use of laboratory animals.

#### 2.3.4. Dietary Protocol

Prior to the experimental phase, all animals were adapted to a low-protein diet (8.8% protein; pellets, 10 mm; Ssniff Spezialdiäten GmbH, E15202–24) for 8 weeks. Following adaptation, a 45-day feeding trial was conducted. The control group continued receiving the low-protein diet, whereas experimental groups were fed the same diet supplemented with 4% (*w*/*w*) dried beetroot (*Boldor* or *Wodan* cultivars, depending on treatment). The experimental diets were prepared from individual ingredients, and their detailed composition is provided in Table S1. The inclusion level corresponded to an estimated intake of approximately 0.71 g beetroot powder per rat per day (~2.05 g/kg body weight/day). Based on body surface area conversion, this dose is equivalent to approximately 23 g/day of dried beetroot for a 70 kg adult, corresponding to ~140 g/day of one fresh beetroot (depending on moisture content). Experimental diets were prepared from individual components. Diets were stored frozen (−40 °C) to preserve stability, thawed weekly, and subsequently kept at 4 °C in sealed containers until use. To minimize bias, investigators responsible for outcome measurements were blinded to group allocation. The entire experimental procedure was independently replicated to confirm reproducibility. At the end of the feeding period, animals were fasted overnight (12 h) prior to sample collection. General anesthesia was induced via intraperitoneal injection of ketamine (100 mg/kg body weight) and xylazine (10 mg/kg body weight). Following confirmation of adequate anesthesia depth, whole blood was collected from the caudal vena cava. Internal organs and thoracic aortae were excised and processed immediately. All animals (n = 10 per group) were included in the sampling procedure.

### 2.4. Methods

#### 2.4.1. Sample Preparation and Microwave Digestion

For elemental analysis, dried and homogenized samples (~0.5 g) were transferred into polytetrafluoroethylene digestion vessels and treated with 5 mL concentrated nitric acid (65% HNO_3_) and 1 mL hydrogen peroxide (30% H_2_O_2_). Mineralization was performed using a microwave digestion system (Ethos One) with a multi-step temperature program, including an initial equilibration phase at room temperature, followed by controlled heating to 200 °C and subsequently to 220 °C, and a final cooling stage. After digestion, samples were diluted to 50 mL with ultrapure water. Procedural blanks were prepared in parallel to verify the absence of contamination.

Elemental concentrations of As, Cd, Cu, Cr, Fe, Ni, Pb, Se, Sb, and Zn were determined using an ICP-MS system (Agilent 7700x, Agilent Technologies, Santa Clara, CA, USA) equipped with a quadrupole analyzer and an Octopole Reaction System (ORS). Instrument parameters were optimized daily and maintained constant throughout the analysis. The ORS was operated in helium collision mode to reduce polyatomic interferences. Rhodium (20 µg/L) was used as an internal standard to correct for signal drift and matrix effects, while residual spectral interferences affecting selenium determination were corrected using mathematical correction procedures. High-purity argon (99.999%) served as both the plasma and carrier gas.

Calibration curves were prepared using multi-element standard solutions over concentration ranges of 1–500 µg/L for Fe and 0.05–100 µg/L for the remaining elements. All calibration curves exhibited excellent linearity (R^2^ > 0.9996). The instrumental operating conditions are presented in Table 1.

#### 2.4.2. Blood Analysis

Biochemical and immunochemical assays were performed using blood serum obtained after centrifugation of blood samples. Biochemical assays were conducted using the BA400 analyzer (BioSystems S.A., Barcelona, Spain). The analyzer employs automated spectrophotometric, enzymatic, colorimetric, and ion-selective electrode (ISE) methods depending on the analyzed parameter. Immunochemical assays were performed using the Cobas e411 system (Roche Diagnostics, Basel, Switzerland), based on electrochemiluminescence immunoassay technology (ECLIA).

#### 2.4.3. Body Composition Analysis (NMR)

Whole-body composition, including lean mass and fat mass, was determined using nuclear magnetic resonance (NMR) with a Minispec (Bruker, Germany). Measurements were performed in conscious animals without the need for anesthesia, according to the manufacturer’s protocol. The instrument was calibrated prior to analysis, and all measurements were conducted under standardized conditions. Measurements were performed at baseline and at the end of the experimental period. Results were expressed as absolute values (g).

#### 2.4.4. Vascular Reactivity Studies

As previously described [6,18], thoracic aortae were excised from rats, immediately placed on ice, and carefully cleaned of surrounding connective tissue. Aortic segments were cut into 4–5 mm rings and mounted in 5 mL static organ baths (Graz Tissue Bath System, Barcelona, Spain) containing Krebs–Henseleit solution (KHS). The baths were continuously aerated with carbogen, and rings were set to a resting tension of 1.5 g using an isometric force transducer system (TAM–A, Hugo Sachs Elektronik, March, Germany).

Vascular functional integrity was confirmed by contractile responses to KCl (75 mM) and endothelium-dependent relaxation to acetylcholine (ACh, 10 μM). After verification, tissues were washed with fresh KHS and allowed to return to baseline tension.

Following a 120 min stabilization period, vascular relaxation was evaluated by constructing cumulative concentration–response curves to acetylcholine chloride (0.1 nM–10 μM) in rings precontracted with noradrenaline (NA, 0.1 μM).

#### 2.4.5. Isolated Perfused Heart (Langendorff Preparation)

As previously described [19], cardiac function was assessed using the Langendorff isolated heart preparation. At the end of the experimental period, rats were anesthetized with ketamine (100 mg/kg) and xylazine (10 mg/kg, i.p.). Hearts were rapidly excised, placed in ice-cold Krebs–Henseleit buffer, and mounted on a Langendorff perfusion system (Hugo Sachs Elektronik, March, Germany) connected to ISOHEART software (version 73–0161).

The hearts were perfused retrogradely via the aorta with Krebs–Henseleit buffer. The perfusate was maintained at 37 °C and continuously aerated with 95% O_2_ and 5% CO_2_.

After a stabilization period of 15–20 min, a fluid-filled latex balloon connected to a pressure transducer was inserted into the left ventricle via the left atrium. Balloon volume was adjusted to obtain a diastolic pressure of 8–10 mmHg. Cardiac parameters recorded during the experiment included heart rate (HR), systolic pressure, diastolic pressure, left ventricular developed pressure (LVDP), maximal rate of pressure development (+dP/dtmax), and coronary flow. Rate pressure product (RPP) was calculated as HR × LVDP and used as an index of cardiac mechanical performance.

### 2.5. Data Analysis and Statistics

For cumulative concentration–response curves (CCRCs), the area under the curve (AUC), maximal response (Emax, %), and potency (logEC_50_) were calculated using nonlinear regression analysis. For isolated perfused heart (IPH) data were recorded using ISOHEART software and analyzed as mean ± SEM. To compare the control group with the four beetroot-supplemented groups, one-way analysis of variance (ANOVA) followed by Dunnett’s multiple-comparisons test was applied. Differences among the beetroot-supplemented groups were evaluated using two-way ANOVA with cultivar and selenium enrichment as fixed factors, followed by Tukey’s multiple-comparisons test. For repeated measurements (before vs. after supplementation), a linear mixed-effects model was applied with time as a within-subject factor and experimental group as a between-subject factor, with individual animals included as a random effect. Changes in the analyzed parameters were calculated as Δ (post − pre). Prior to analysis, data were screened for normality and potential outliers. Data are expressed as mean ± standard deviation (SD) or mean ± standard error of the mean (SEM) for CCRCs and IPH. The experiment was arranged in a randomized complete block design (RCBD) with four replicates. All statistical analyses were performed using GraphPad Prism 11.0.1 (San Diego, CA, USA). Effect size was evaluated using Cohen’s d, calculated as the standardized difference between group means. Effect sizes were interpreted according to conventional thresholds (small: 0.2, medium: 0.5, large: 0.8). Statistical significance was set at *p* < 0.05.

## 3. Results

At the end of the acclimatization period (8 weeks), rat body weights were similar across all groups (Table 2).

### 3.1. Groups Were Analyzed Separately, Although Dietary Metal Content Was Similar

Foliar application of the selenium-based plant growth stimulator did not result in a statistically significant dose-dependent increase in selenium (Se) or in the analyzed trace elements and potentially toxic elements (Fe, Zn, Cu, Cr, Ni, Pb, As, Cd, and Sb) in beet roots (Supplementary Table S2).

Although the diets contained similar levels of trace elements and potentially toxic elements, the experimental groups were analyzed separately to evaluate the specific effects of the individual preparations and foliar application of the selenium-based plant growth stimulator.

Body weight increased significantly over the course of the experimental period in all groups (*p* < 0.001), while baseline values did not differ between treatments. Among the dietary interventions, the *Boldor* 1 group exhibited a significantly greater body weight gain compared with the control group (*p* = 0.009), accompanied by a large effect size (Cohen’s d = 1.05). In contrast, the *Wodan* 3 and *Boldor* 3 groups showed only a tendency toward increased weight gain relative to the control. Overall, the pattern of mean body weight changes followed the order: *Boldor* 1 > *Wodan* 3 > *Boldor* 3 > *Wodan* 1 > control (see Table 2).

Lean mass increased significantly over time in all groups (*p* < 0.001). However, no significant differences in lean mass gain were observed between the experimental groups. The *Wodan* 3 group (*p* = 0.077) and the *Boldor* 3 group (*p* = 0.061) showed a tendency toward higher lean mass gain. The trend in mean changes followed the order: *Wodan* 3 > *Boldor* 3 > *Boldor* 1 > *Wodan* 1 > control (Table 3).

Body fat mass increased significantly over the experimental period in all groups (*p* < 0.001). Notably, the *Boldor* 1 group exhibited higher fat mass gain compared with the control, approaching statistical significance (*p* = 0.05), indicating a clear upward trend. No statistically significant differences were detected among the remaining experimental groups. The pattern of mean fat mass gain followed the order: *Boldor* 1 > *Wodan* 3 > *Boldor* 3 > *Wodan* 1 ≈ control (Table 4).

Average daily feed intake did not differ significantly between experimental groups (ANOVA, *p* = 0.293), and no differences were observed between supplemented groups and the control group. The trend in mean values was *Boldor* 1 > *Wodan* 3 > *Boldor* 3 > *Wodan* 1 > control (Table 5).

No significant differences in liver, heart, spleen, or kidney weights were observed between the experimental groups (ANOVA, *p* > 0.05). The mean organ weights showed the following order: liver (*Boldor* 1 ≈ *Wodan* 3 > *Boldor* 3 > control > *Wodan* 1), heart (*Wodan* 3 > *Boldor* 3 > *Boldor* 1 > *Wodan* 1 > control), spleen (*Wodan* 1 > *Wodan* 3 > *Boldor* 3 > *Boldor* 1 > control), and kidney (*Boldor* 3 > *Wodan* 1 > *Boldor* 1 > *Wodan* 3 > control) (Table 6).

Highly significant differences were observed for 14 of the 30 measured biochemical parameters, namely total bilirubin, sodium, lipase, CRP (C-reactive protein), albumin, chloride, iron, total protein, amylase, calcium, ALP (alkaline phosphatase), ALT (alanine aminotransferase), and potassium concentrations among the experimental groups (Table 7). Among all analyzed biochemical parameters, total bilirubin exhibited the strongest statistical effect, whereas potassium showed the weakest, although still statistically significant, effect.

Collectively, these findings suggest that beetroot supplementation may modulate hepatic metabolism, inflammatory status, oxidative balance, and electrolyte homeostasis in rats maintained on a low-protein diet. The observed increase in bilirubin may also be biologically relevant due to its recognized antioxidant properties.

The *Wodan* group was characterized by significantly higher blood potassium concentrations compared with the *Boldor* and control groups. In contrast, lipase activity was significantly lower in the *Wodan* group than in the other study groups.

Neither the lipid profile parameters, renal function biomarkers, nor cardiac and muscle injury markers showed significant differences between the experimental groups. These findings suggest that beetroot supplementation did not adversely affect lipid metabolism, kidney function, or muscle and cardiac tissue integrity under the applied experimental conditions.

### 3.2. Pooling of Boldor and Wodan Groups Based on Comparable Levels of Trace Elements and Potentially Toxic Elements in the Diets

Initially, animals were assigned to four groups (*Boldor* 1, *Boldor* 3, *Wodan* 1, and *Wodan* 3). As no differences in the levels of trace elements and potentially toxic elements were observed between the corresponding diets (Table S2), the groups were pooled for statistical analysis, resulting in two combined groups: *Boldor* and *Wodan*.

**Figure 1:**
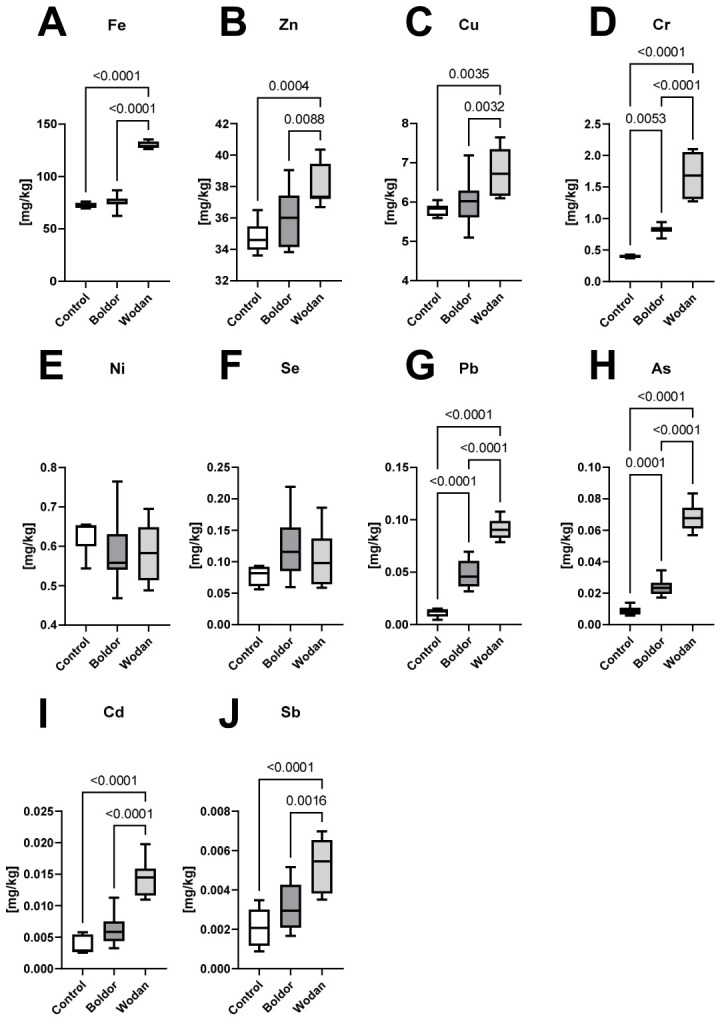
Concentrations of selected trace elements (Fe, Zn, Cu, Cr, Ni, Se) and potentially toxic elements (Pb, As, Cd, Sb) in experimental diets across treatment groups (**A**–**J**).

The highest concentrations of the analyzed elements (Fe, Zn, Cu, Cr, Pb, As, Cd, and Sb) were observed in the *Wodan* treatment compared with both the control and *Boldor* diets. No significant differences between the control vs. *Boldor* diets were detected for Fe, Zn, Cu, Cd, and Sb (*Boldor* = control). However, the *Boldor* diet exhibited significantly higher concentrations of Cr, Pb, and As relative to the control (*Boldor* > control). Furthermore, no significant differences were found among the *Wodan*, *Boldor*, and control treatments for Ni and Se (*Wodan* = *Boldor* = control), see Figure 1.

**Figure 2:**
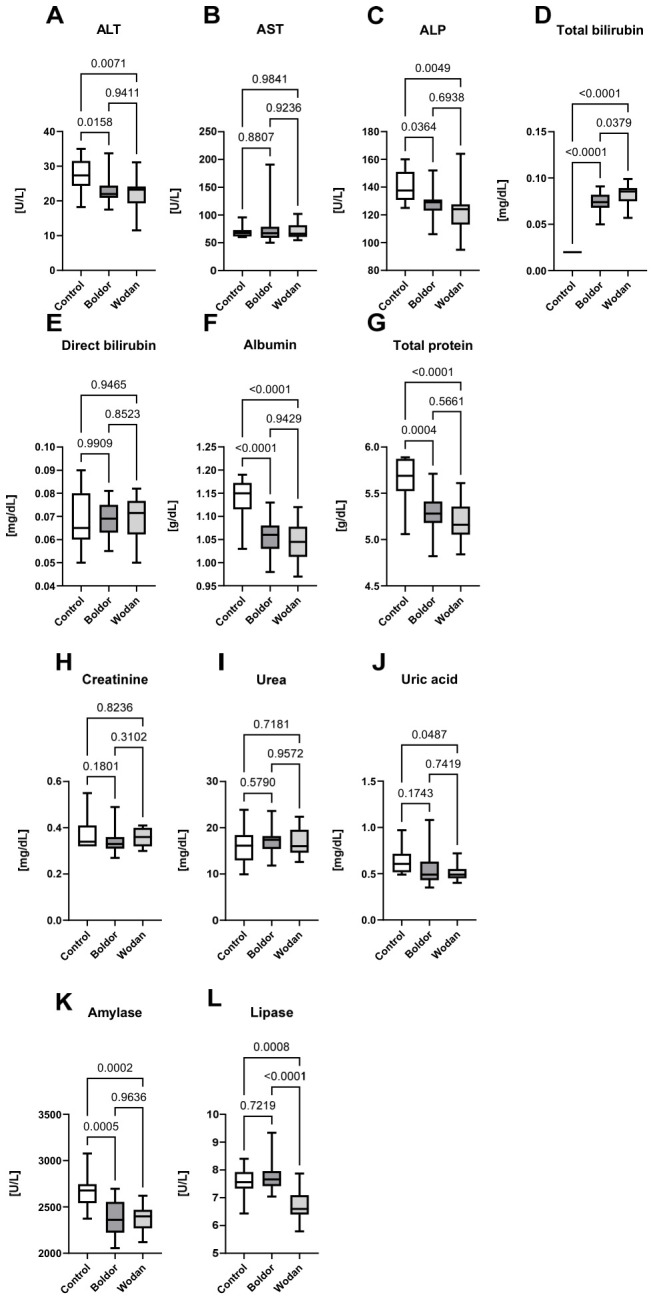

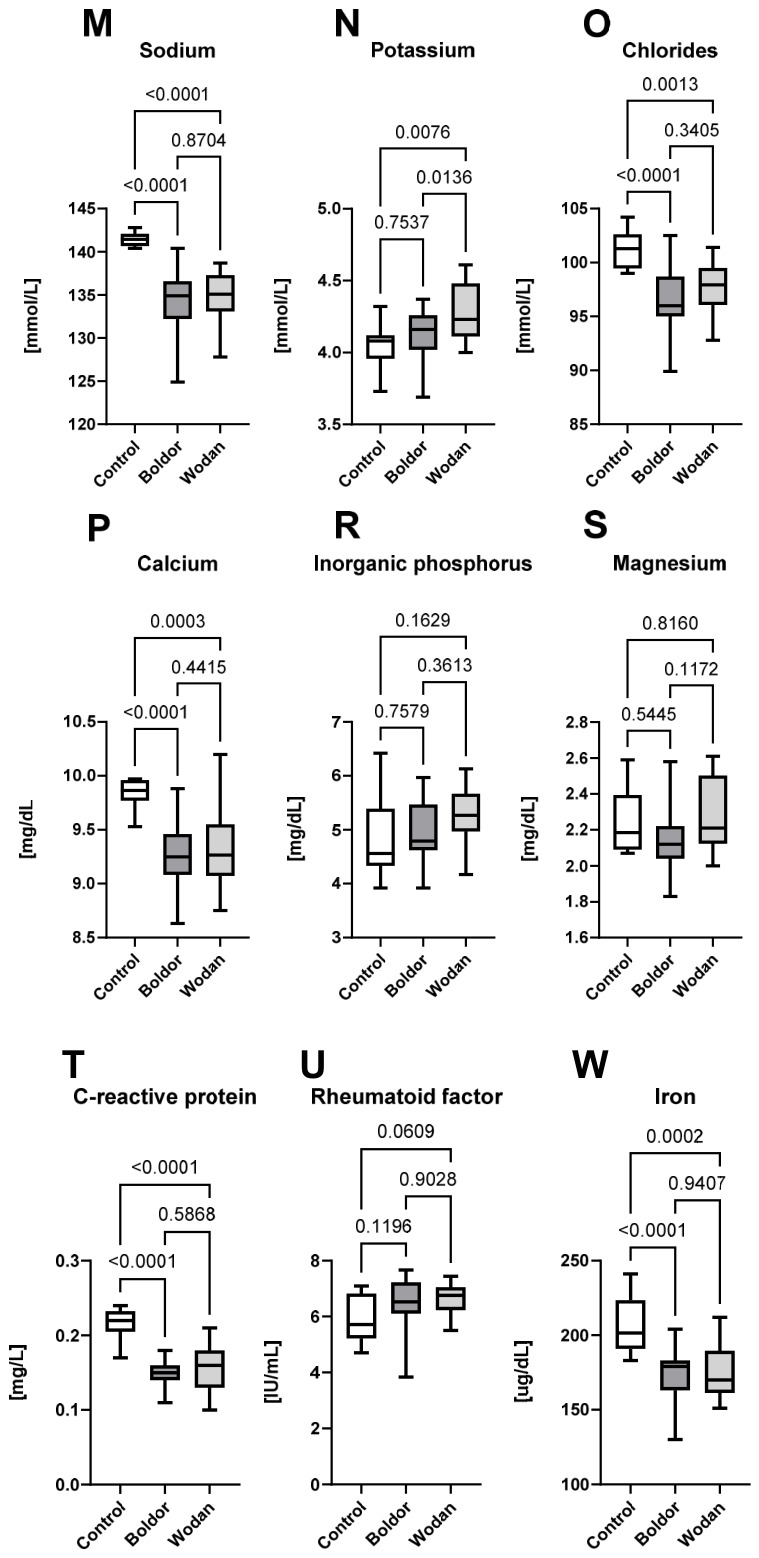
Blood serum biomarkers of hepatic function (**A**–**G**), renal function (**H**–**J**), pancreatic enzyme activity (**K**,**L**), electrolyte homeostasis (**M**–**O**), mineral metabolism (**P,R,S**), inflammatory and immunological status (**T**,**U**), and nutritional metabolism (**W**). Data were analyzed by one-way ANOVA with Tukey’s multiple-comparisons test. Significant differences between groups are indicated in the figure (*p* < 0.05).

Beetroot supplementation modulated a broad spectrum of biochemical indices, affecting biomarkers of liver function (ALT, ALP, total bilirubin, albumin, and total protein), renal function (uric acid), pancreatic activity (amylase and lipase), electrolyte balance (sodium, potassium, and chloride), mineral metabolism (calcium, inorganic phosphorus, and magnesium), inflammatory status (CRP), and nutritional metabolism (iron), see Figure 2A–P,R–U,W. Conversely, no significant effects were observed on lipid profile parameters or biomarkers of cardiac and skeletal muscle injury, see Supplementary Materials (Figure S1). Among the beetroot cultivars evaluated, *Wodan* exerted distinct effects relative to *Boldor*, resulting in higher circulating total bilirubin (Figure 2D) and potassium concentrations (Figure 2N), alongside reduced uric acid (Figure 2J) and lipase (Figure 2L) levels in treated rats.

Body weight increased significantly over time in all groups (*p* < 0.001). The *Boldor* group exhibited greater body weight gain compared with the control group (*p* = 0.020), with a moderate-to-large effect size (Cohen’s d = 0.70–0.89), whereas the *Wodan* group did not differ significantly from the control. The overall pattern of mean body weight changes followed the order: *Boldor* > *Wodan* > control.

Lean mass also increased significantly during the experimental period (*p* < 0.001); however, no statistically significant differences in lean mass gain were observed between the experimental groups and the control, despite a consistent trend of *Boldor* > *Wodan* > control.

Similarly, fat mass increased over time (*p* < 0.001), but no significant between-group differences were detected. Nevertheless, the pattern of mean changes was comparable, with values ordered as *Boldor* > *Wodan* > control (Table 8).

No significant differences in liver or heart weights were observed between the groups. Spleen weight was higher in the *Wodan* group compared with the control, with a large effect size (Cohen’s d = 0.83). Kidney weight was increased in both experimental groups (*Boldor* and *Wodan*) relative to the control. Effect size analysis revealed a large difference between the control and *Boldor* groups (Cohen’s d = 0.86) and a moderate-to-large difference between the control and *Wodan* groups (Cohen’s d = 0.70), whereas the difference between *Boldor* and *Wodan* was small (Cohen’s d = 0.17).

The trends in mean organ weights were as follows: liver (*Boldor* > control > *Wodan*), heart (*Wodan* > *Boldor* > control), spleen (*Wodan* > *Boldor* > control), and kidney (*Boldor* > *Wodan* > control) (Table 9).

Average daily feed intake did not differ significantly between groups (ANOVA, *p* = 0.234), indicating comparable intake relative to the control. The trend in mean values followed the order: *Boldor* > *Wodan* > control (Table 10).

Feed efficiency ratio was highest in the *Boldor* group, indicating greater body weight gain relative to feed intake compared with the control; however, this difference did not reach statistical significance (ANOVA, *p* = 0.117; post hoc test, *p* = 0.063). The *Wodan* group showed intermediate values (*p* = 0.446), resulting in the following trend: *Boldor* > *Wodan* > control. Notably, the higher feed efficiency observed in the *Boldor* group occurred despite feed intake being comparable to that of the control group (Table 10).

#### 3.2.1. Vascular Relaxation and Contraction

**Figure 3:**
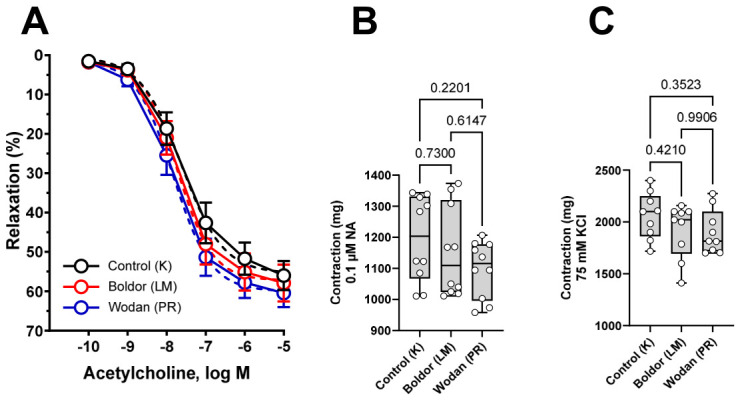
Vasodilatory responses to acetylcholine (0.1 nM–10 μM, (**A**)) and vasoconstrictor responses to noradrenaline (0.1 μM, (**B**,**C**)) and KCl (75 mM) in isolated aortic rings. Control (K), *Boldor* (LM), *Wodan* (PR). *p* > 0.05, ANOVA. Dashed lines indicate the four-parameter logistic (4PL) nonlinear regression fits to the concentration–response data.

Vascular responses of isolated aortic rings were not affected by contraction induced by noradrenaline (NA, 0.1 μM) or KCl (75 mM), nor by relaxation in response to cumulative concentrations of acetylcholine (0.1 nM–10 μM) (Figure 3 and Table 11).

#### 3.2.2. The Isolated Perfused Heart

In the Langendorff isolated heart assay, *Boldor* and *Wodan* treatments did not significantly affect cardiac contractility, heart rate, coronary flow, or overall cardiac performance compared with control (Table 12).

## 4. Discussion

Elemental analysis revealed that the highest concentrations of Fe, Zn, Cu, Cr, Pb, As, Cd, and Sb were observed in the *Wodan* treatment compared with both control and *Boldor* groups. No significant differences between control and *Boldor* were detected for Fe, Zn, Cu, Cd, and Sb, while Cr, Pb, and As were elevated in *Boldor* relative to control. In contrast, Ni and Se levels did not differ among treatments, indicating similar accumulation patterns. While *Wodan* exhibited the highest concentrations of several essential trace elements, it also accumulated greater amounts of potentially toxic elements, including Pb, As, Cd, and Sb, suggesting that enhanced mineral uptake may be accompanied by reduced selectivity of elemental acquisition.

Foliar application of the selenium-based plant growth stimulator did not result in a statistically significant dose-dependent increase in selenium (Se) or in the analyzed elements. These findings point to a cultivar-dependent accumulation profile, likely reflecting differences in mineral uptake and translocation mechanisms. This interpretation is consistent with previous reports demonstrating that trace element accumulation depends on genotype, environmental conditions, and selenium form [20,21], as well as with studies documenting cultivar-specific mineral variability in beetroot under biofortification conditions [22].

Although studies directly linking beetroot cultivar differences with metabolic efficiency in animal models are limited, beetroot is recognized as a rich source of biologically active compounds that may influence energy metabolism and mitochondrial function [23,24]. Furthermore, selenium application has been shown to enhance plant nutritional quality and increase the concentration of bioactive metabolites, potentially amplifying physiological effects [22].

Highly significant differences were observed for 14 of the 30 measured biochemical parameters, including alanine aminotransferase (ALT), alkaline phosphatase (ALP), total bilirubin, albumin, total protein, uric acid, amylase, lipase, sodium, potassium, chloride, calcium, C-reactive protein (CRP), and iron concentrations. Among these variables, total bilirubin exhibited the most pronounced statistical effect. The increase in bilirubin may be biologically relevant given its well-established antioxidant and cytoprotective properties. Collectively, these findings suggest that beetroot supplementation influences hepatic metabolism, inflammatory status, redox homeostasis, and electrolyte balance in rats maintained on a low-protein diet.

Beetroot supplementation did not improve lipid profile parameters or biomarkers of cardiac and skeletal muscle injury in the experimental groups, which may partly explain the lack of beneficial effects on ex vivo cardiac function and thoracic artery vascular reactivity observed in the present study.

Interestingly, although total bilirubin concentration differed significantly between groups, no significant differences were observed for direct bilirubin. This suggests that the dietary intervention predominantly affected the unconjugated bilirubin fraction rather than hepatic conjugation or biliary excretion processes. The absence of changes in direct bilirubin may indicate preserved liver excretory function despite alterations in overall bilirubin metabolism. These findings may be associated with the antioxidant properties of beetroot-derived bioactive compounds, which could influence heme metabolism and oxidative balance.

The increase in total bilirubin concentration may also be biologically relevant because bilirubin is recognized as an endogenous antioxidant. Moderate elevations in bilirubin have been associated with enhanced antioxidant capacity and protection against oxidative stress-related cellular damage [25,26,27]. Therefore, the observed alterations in bilirubin metabolism may reflect adaptive antioxidant responses induced by beetroot-derived bioactive compounds.

The observed reduction in amylase and lipase activity in beetroot-supplemented groups may indicate a modulatory effect of beetroot-derived bioactive compounds on pancreatic function and digestive metabolism. Beetroot is rich in betalains, polyphenols, and dietary fiber, which may reduce metabolic stress and improve gastrointestinal homeostasis [28]. The concomitant decrease in inflammatory markers, particularly CRP, suggests that lower pancreatic enzyme activity may be associated with reduced systemic inflammation and oxidative stress rather than impaired pancreatic function. Furthermore, adaptation to a low-protein diet combined with beetroot supplementation may have contributed to altered digestive enzyme secretion and metabolic regulation.

The reductions in serum sodium and chloride concentrations observed in beetroot-supplemented groups may be related to the high nitrate content of beetroot and the consequent increase in nitric oxide bioavailability [28,29,30]. Nitric oxide promotes renal vasodilation and natriuresis, and may also facilitate uric acid excretion, potentially contributing to the lower circulating uric acid concentrations observed in supplemented animals. Nevertheless, the mechanisms underlying these changes require further investigation. The decrease in calcium concentration may additionally result from altered mineral absorption and binding interactions involving beetroot-derived oxalates and polyphenolic compounds. The oxalate content of beetroot root is approximately 0.5% of dry matter [31]. Oxalates form insoluble calcium oxalate salts, reducing calcium bioavailability and intestinal absorption. This mechanism may have contributed to the observed changes in plasma calcium concentration. Nevertheless, beetroot is characterized by a relatively low oxalate content—approximately 15-fold lower than that of raw spinach [31]—and is therefore unlikely to substantially affect calcium homeostasis. Therefore, the observed changes in plasma calcium levels were likely also influenced by other metabolic mechanisms or dietary factors. Furthermore, significant differences in albumin concentration between groups may have influenced total serum calcium values, as a substantial fraction of circulating calcium is protein-bound. Overall, these findings likely reflect adaptive metabolic and electrolyte changes rather than overt pathological disturbances.

Alkaline phosphatase activity was significantly reduced in beetroot-supplemented groups compared with the control animals. Since elevated alkaline phosphatase is commonly associated with hepatobiliary stress and inflammatory processes, the observed decrease may indicate a protective effect of beetroot-derived bioactive compounds on liver metabolism [32]. The concomitant reduction in ALT, CRP and the absence of significant changes in direct bilirubin further support the interpretation that beetroot supplementation may alleviate hepatic and systemic inflammatory burden rather than induce cholestatic alterations [33]. Additionally, changes in mineral metabolism observed under low-protein dietary conditions may have partially contributed to alterations in alkaline phosphatase activity.

A significant reduction in serum iron concentration was observed in beetroot-supplemented groups compared with the control animals. This finding may reflect alterations in iron metabolism associated with the biological activity of beetroot-derived compounds. Beetroot is rich in polyphenols and betalains, which may influence iron absorption, transport, and tissue distribution. Additionally, the reduction in inflammatory markers observed in the supplemented groups suggests that modulation of inflammatory pathways and iron homeostasis may have contributed to lower circulating iron concentrations. Another possible explanation is increased utilization of iron for erythropoietic or metabolic processes rather than a true systemic iron deficiency.

The differences observed between *Wodan* and *Boldor* indicate that cultivar selection may influence the metabolic effects of beetroot supplementation. Despite belonging to the same species, beetroot cultivars can vary substantially in their phytochemical and mineral composition, including betalains, phenolics, nitrates, and potassium. Such differences may account for the higher bilirubin and potassium concentrations and lower uric acid and lipase levels observed in *Wodan*-treated animals. Although the underlying mechanisms remain unclear, these findings suggest that cultivar-specific characteristics contribute to the variability of physiological responses to beetroot consumption and should be considered in future nutritional and functional food studies.

The compositional differences between cultivars were also associated with distinct metabolic outcomes. *Boldor* supplementation increased body weight gain without affecting feed intake, indicating improved feed efficiency. This effect was supported by an increased feed efficiency ratio, suggesting enhanced nutrient utilization rather than increased energy intake. Under protein-restricted conditions, such adaptations may reflect improved metabolic efficiency, potentially involving enhanced mitochondrial function, nitrogen utilization, or energy partitioning [34]. Beetroot contains bioactive compounds such as betalains, nitrates, polyphenols, and trace minerals, which may contribute to these effects.

Body composition analysis confirmed that both lean and fat mass increased over time in all groups, reflecting normal growth. However, treatment-related differences were limited. Interestingly, this was the *Boldor* 1 group (*Boldor* with no AminoSelenit application) which exhibited significantly higher body weight gain and fat mass, along with a tendency toward greater lean mass gain, whereas *Wodan* supplementation produced only minor changes. These findings suggest that *Boldor* may promote a more pronounced anabolic response under protein restriction. Importantly, the increase in body weight was not solely attributable to adiposity, indicating a combined effect on both lean and fat compartments, consistent with improved nutrient partitioning and metabolic adaptation.

Foliar application of the selenium-based plant growth stimulator and beet-derived bioactive compounds may have contributed to these metabolic effects. Selenium plays a central role in antioxidant defense through its incorporation into selenoproteins such as glutathione peroxidases, which regulate redox balance and influence metabolic processes [20,21]. In parallel, beetroot-derived antioxidants, including betalains, may synergistically enhance oxidative stability [35]. Selenium application has also been shown to improve plant biochemical composition, including increased levels of proteins, soluble sugars, and antioxidant compounds [22,36], highlighting a close interaction between selenium metabolism and plant biochemistry [37].

Organ weight analysis provided additional insight into physiological responses. Both *Boldor* and *Wodan* increased kidney weight, whereas spleen weight was elevated only in the *Wodan* group. Increased kidney mass may reflect adaptive responses related to altered nitrogen metabolism or increased renal workload under protein restriction [38]. The higher spleen weight observed in the *Wodan* group suggests that this cultivar may have exerted additional physiological effects beyond those observed for *Boldor*. Selenium and plant-derived phytochemicals have been reported to influence immune responses and redox homeostasis [1,21,39]; therefore, these mechanisms may potentially contribute to the observed organ-specific responses.

Despite the observed metabolic and organ-level changes, no significant differences were detected in vascular reactivity or cardiac function. Parameters derived from both isolated aortic rings and the Langendorff heart model remained unchanged across all groups. This lack of cardiovascular effects may be attributed to the moderate level of dietary supplementation, the relatively short duration of the study, or the use of normotensive animals. While dietary nitrates from beetroot are known to exert vasodilatory effects via nitric oxide-mediated pathways [1], the experimental conditions applied here may have been insufficient to elicit detectable hemodynamic changes.

The stability and bioavailability of beetroot bioactive compounds may also influence these outcomes, as processing conditions can affect antioxidant capacity and phytochemical composition [40], potentially contributing to variability in physiological responses.

The accumulation of potentially toxic elements, particularly Cd, Pb, and As, in the *Wodan* cultivar warrants careful consideration. Although no overt toxicological effects were observed in the present study, the elevated concentrations of these elements highlight the importance of considering food safety aspects when evaluating beetroot cultivars. These findings underscore the need to account for cultivar-dependent variability in elemental composition alongside potential nutritional and physiological benefits [3,8,10,41]. From the perspective of functional food development, the results emphasize the importance of a balanced evaluation of beetroot cultivars, taking into account both their health-promoting properties and their elemental composition.

Interestingly, while agronomic studies frequently report improvements in plant composition following application of the selenium-based plant growth stimulator [22], such compositional changes do not necessarily translate into measurable physiological effects, particularly in cardiovascular parameters. Similar dissociations between compositional and functional outcomes have been reported in crop studies, where fertilization strategies altered biochemical composition without affecting yield [42]. This underscores the complexity of translating plant biofortification into systemic biological responses.

Several limitations of this study should be acknowledged. First, the experiment was conducted in normotensive WKY rats, which may have limited the detection of cardiovascular effects that could be more pronounced in disease models, such as hypertensive animals. Nevertheless, the low-protein diet induced measurable metabolic and physiological alterations, confirming that the dietary intervention affected the animals’ health status and provided a relevant model for evaluating the potential effects of beetroot supplementation. Second, the supplementation period was relatively short, and longer-term interventions may reveal additional physiological adaptations. Third, mechanistic investigations were beyond the scope of the present study and therefore the biological pathways underlying the observed effects remain to be elucidated. Fourth, the beetroot cultivars were evaluated following cultivation in a single location during one growing season. Consequently, the observed cultivar-related differences may have been influenced by environmental factors and genotype × environment interactions. Future multi-location and multi-year studies are needed to confirm the stability and reproducibility of the genotype-specific effects observed in the present work. Fifth, the plant material from replicate plots was pooled prior to diet preparation, preventing the use of independent diet replicates in the feeding experiment. Finally, parameters such as nitrate content, betalain concentration, polyphenol profile, and antioxidant capacity were not determined. As these compounds are considered major contributors to the biological activity of beetroot, their absence limits the mechanistic interpretation of the observed cultivar-dependent effects.

## 5. Conclusions

Beetroot supplementation influenced metabolic, biochemical, and organ-specific responses in rats fed a low-protein diet, with effects depending on cultivar type. *Boldor* primarily improved growth performance and feed efficiency, whereas *Wodan* produced more pronounced changes in biochemical markers associated with antioxidant status, mineral metabolism, and inflammatory regulation. Neither cultivar significantly affected vascular reactivity or cardiac function under the experimental conditions. These findings demonstrate that beetroot cultivar selection is an important determinant of physiological outcomes and should be considered when evaluating the functional properties of beet-based dietary interventions. Further studies are needed to elucidate the underlying mechanisms and assess the long-term implications of these cultivar-specific effects.

## Figures and Tables

**Table 1:** Instrumental operating conditions for ICP-MS analysis.

Parameter	Setting
Instrument	ICP-MS 7700x (Agilent Technologies, Santa Clara, CA, USA)
Cones	Ni
RF Power (W)	1550
Nebulizer gas flow (L/min)	0.98
Auxiliary gas flow (L/min)	0.90
Plasma gas flow (L/min)	15.50
Peristaltic pump speed (rps)	0.10
He flow (mL/min)	6.0 (Cu, Zn); 4.0 (Cr, Fe, Ni, Sb, Se) 3.0 (As, Cd, Pb)
Monitored isotopes	^75^As, ^111^Cd, ^52^Cr, ^65^Cu, ^56^Fe, ^60^Ni, ^123^Sb, ^82^Se, ^208^Pb, ^68^Zn
Integration time per *m*/*z* (s)	0.1
Replicates	3
Sweeps per replicate	100

The equation used for signal correction was as follows: Mc (82) = 1.0000 × M (82) − 1.0078 × M (83).

**Table 2:** Body weight gain (Δ) in the control and experimental groups of rats.

Group	Before (Mean ± SD)	After (Mean ± SD)	Δ (Mean ± SD)	LMPR vs. K (Mixed Model)
Control (K)	302.0 ± 23.17	382.8 ± 28.74	80.80 ± 13.05	–
*Boldor* 1 (L)	302.0 ± 19.07 ^a^	395.4 ± 19.76 ^a^	93.38 ± 10.74 ^a^	0.009 *
*Boldor* 3 (M)	303.2 ± 22.81 ^a^	390.0 ± 14.94 ^ab^	86.81 ± 11.36 ^a^	0.134
*Wodan* 1 (P)	302.3 ± 19.47 ^a^	384.0 ± 18.06 ^b^	81.68 ± 7.76 ^a^	0.855
*Wodan* 3 (R)	302.0 ± 21.22 ^a^	394.6 ± 16.15 ^ab^	89.79 ± 12.49 ^a^	0.069
Cultivar effect	-	*p* = 0.049	*p* = 0.19	
Se effect	-	*p* = 0.701	*p* = 0.63	
Cultivar x Se	-	*p* = 0.891	*p* = 0.10	

Data are presented as mean ± standard deviation (SD). * *p* ≤ 0.05. Δ represents the difference between post-supplementation and baseline values. Means with the same letter(s) are not significantly different (*p* > 0.05). Means with different letters are significantly different (*p* ≤ 0.05). The pattern of mean body weight changes followed the order: *Boldor* 1 > *Wodan* 3 > *Boldor* 3 > *Wodan* 1 > control.

**Table 3:** Changes in lean mass (Δ) assessed by NMR after supplementation in rats.

Group	Before (Mean ± SD)	After (Mean ± SD)	Δ (Mean ± SD)	LMPR vs. K (Mixed Model)
Control (K)	211.69 ± 18.34	258.47 ± 17.28	46.78 ± 4.32	–
*Boldor* 1 (L)	211.80 ± 15.24 ^a^	264.77 ± 16.33 ^a^	52.97 ± 5.12 ^a^	0.148
*Boldor* 3 (M)	214.91 ± 13.91 ^a^	269.67 ± 11.52 ^a^	54.76 ± 6.62 ^a^	0.061
*Wodan* 1 (P)	214.86 ± 16.11 ^a^	265.97 ± 10.93 ^a^	51.11 ± 9.84 ^a^	0.317
*Wodan* 3 (R)	211.76 ± 15.82 ^a^	267.90 ± 11.30 ^a^	56.14 ± 6.39 ^a^	0.077
Cultivar effect	-	*p* = 0.83	*p* = 0.76	
Se effect	-	*p* = 0.96	*p* = 0.27	
Cultivar x Se	-	*p* = 0.64	*p* = 0.15	

Data are presented as mean ± standard deviation (SD). Means with the same letter(s) are not significantly different (*p* > 0.05). Means with different letters are significantly different (*p* ≤ 0.05). Δ represents the difference between post-supplementation and baseline values. The pattern of lean mass gain followed the order: *Wodan* 3 > *Boldor* 3 > *Boldor* 1 > *Wodan* 1 > control.

**Table 4:** Changes in fat mass (Δ) assessed by NMR after supplementation in rats.

Group	Before (Mean ± SD)	After (Mean ± SD)	Δ (Mean ± SD)	LMPR vs. K (Mixed Model)
Control (K)	20.77 ± 2.73	36.69 ± 6.31	15.93 ± 4.79	–
*Boldor* 1 (L)	20.34 ± 3.35 ^a^	42.67 ± 9.57 ^a^	22.33 ± 10.81 ^a^	0.05
*Boldor* 3 (M)	19.75 ± 3.78 ^a^	37.34 ± 8.28 ^a^	17.59 ± 8.68 ^a^	0.631
*Wodan* 1 (P)	19.42 ± 2.26 ^a^	35.24 ± 6.25 ^a^	15.82 ± 5.34 ^a^	0.975
*Wodan* 3 (R)	22.44 ± 4.24 ^a^	41.61 ± 7.86 ^a^	18.54 ± 7.48 ^a^	0.463
Cultivar effect	-	*p* = 0.46	*p* = 0.79	
Se effect	-	*p* = 0.80	*p* = 0.88	
Cultivar x Se	-	*p* = 0.29	*p* = 0.21	

Data are presented as mean ± standard deviation (SD). Means with the same letter(s) are not significantly different (*p* > 0.05). Means with different letters are significantly different (*p* ≤ 0.05). Δ represents the difference between post-supplementation and baseline values. The pattern of mean fat mass gain followed the order: *Boldor* 1 > *Wodan* 3 > *Boldor* 3 > *Wodan* 1 ≈ control.

**Table 5:** Average daily feed intake (g/day) in rats during the experimental period.

Group	Feed Intake (g/day, Mean ± SD)	LMPR vs. K
Control (K)	17.33 ± 1.11	–
*Boldor* 1 (L)	18.03 ± 0.73 ^a^	0.45
*Boldor* 3 (M)	17.63 ± 0.53 ^a^	0.90
*Wodan* 1 (P)	17.51 ± 0.63 ^a^	0.90
*Wodan* 3 (R)	17.80 ± 0.54 ^a^	0.87
Cultivar effect	*p* = 0.28	
Se effect	*p* = 0.83	
Cultivar x Se	*p* = 0.11	

Data are presented as mean ± standard deviation (SD). *p* > 0.05. LMPR vs. K (one-way ANOVA with Dunnett’s multiple-comparisons test). Different superscript letters indicate significant differences among groups, whereas identical letters indicate no significant differences (two-way ANOVA with Tukey’s multiple comparisons test). Average daily feed intake was calculated as total feed consumption divided by the number of feeding days. The trend in mean values followed the order: *Boldor* 1 > *Wodan* 3 > *Boldor* 3 > *Wodan* 1 > control.

**Table 6:** Organ weights in experimental groups of rats (g).

Group	Liver (mean ± SD)	*p*	Heart (mean ± SD)	*p*	Spleen (mean ± SD)	*p*	Kidney (mean ± SD)	LMPR vs. K
Control (K)	10.75 ± 1.29	–	1.25 ± 0.20	–	0.692 ± 0.100	–	2.02 ± 0.16	–
*Boldor* 1 (L)	11.18 ± 1.02 ^a^	0.74	1.28 ± 0.11 ^a^	0.98	0.716 ± 0.083 ^a^	0.98	2.17 ± 0.18 ^a^	0.30
*Boldor* 3 (M)	10.85 ± 1.18 ^a^	0.99	1.34 ± 0.20 ^a^	0.69	0.736 ± 0.066 ^a^	0.79	2.19 ± 0.22 ^a^	0.26
*Wodan* 1 (P)	10.16 ± 0.47 ^a^	0.61	1.26 ± 0.21 ^a^	0.99	0.773 ± 0.086 ^a^	0.22	2.18 ± 0.09 ^a^	0.22
*Wodan* 3 (R)	11.18 ± 0.67 ^a^	0.69	1.36 ± 0.17 ^a^	0.59	0.761 ± 0.070 ^a^	0.33	2.10 ± 0.19 ^a^	0.86
Cultivar effect	*p* = 0.172		*p* = 0.77		*p* = 0.19		*p* = 0.16	
Se effect	*p* = 0.295		*p* = 0.18		*p* = 0.99		*p* = 0.63	
Cultivar x Se	*p* = 0.731		*p* = 0.11		*p* = 0.17		*p* = 0.83	

Data are presented as mean ± standard deviation (SD). *p* > 0.05. LMPR vs. K (one-way ANOVA with Dunnett’s multiple-comparisons test). Different superscript letters indicate significant differences among groups, whereas identical letters indicate no significant differences (two-way ANOVA with Tukey’s multiple comparisons test). Organ weights were measured at the end of the experimental period. No significant differences in liver, heart, spleen, or kidney weights were observed between the experimental groups.

**Table 7:** Blood analysis.

Assay	Control (K)	*Boldor* 1 (L)	*Boldor* 3 (M)	*Wodan* 1 (P)	*Wodan* 3 (R)	LMPR vs. K
Liver function biomarkers (5/7 affected)						
Alanine aminotransferase (ALT) [U/L]	27.57 ± 5.083	23.91 ± 4.326 ^a^*	21.56 ± 2.136 ^a^*	21.82 ± 5.412 ^a^*	22.87 ± 3.148 ^a^*	* *p* ≤ 0.0311
Aspartate aminotransferase (AST) [U/L]	69.73 ± 10.28	70.81 ± 11.88 ^a^	63.83 ± 10.81 ^a^	73.50 ± 12.59 ^a^	68.76 ± 13.90 ^a^	*p* > 0.7529
Alkaline phosphatase (ALP) [U/L]	139.8 ± 11.53	131.8 ± 11.20 ^a^	125.0 ± 13.49 ^a^*	116.3 ± 11.77 ^a^*	129.6 ± 15.24 ^a^	* *p* > 0.0017
Total bilirubin [mg/dL]	0.020 ± 0.011	0.075 ± 0.013 ^a^*	0.074 ± 0.007 ^a^*	0.081 ± 0.014 ^a^*	0.085 ± 0.009 ^a^*	* *p* < 0.0001
Direct bilirubin [mg/dL]	0.069 ± 0.013	0.0719 ± 0.008 ^a^	0.065 ± 0.006 ^a^	0.068 ± 0.011 ^a^	0.072 ± 0.005 ^a^	*p* > 0.5492
Albumin [g/dL]	1.139 ± 0.048	1.068 ± 0.042 ^a^*	1.032 ± 0.035 ^a^*	1.040 ± 0.041 ^a^*	1.053 ± 0.045 ^a^*	* *p* ≤ 0.0055
Total protein [g/dL]	5.653 ± 0.264	5.369 ± 0.205 ^a*^	5.154 ± 0.239 ^a^*	5.150 ± 0.199 ^a^*	5.230 ± 0.233 ^a^*	* *p* ≤ 0.0429
Lipid profile (0/5 affected)						
Total cholesterol [mg/dL]	99.29 ± 9.923	96.65 ± 9.400 ^a^	87.94 ± 6.744 ^a^	95.60 ± 6.362 ^a^	95.86 ± 11.22 ^a^	*p* > 0.0674
HDL cholesterol [mg/dL]	68.00 ± 7.535	66.56 ± 7.621 ^a^	58.73 ± 7.114 ^a^	64.41 ± 4.647 ^a^	64.71 ± 8.943 ^a^	*p* > 0.0639
LDL cholesterol [mg/dL]	14.41 ± 1.635	11.53 ± 1.970 ^a^	12.44 ± 1.497 ^a^	13.17 ± 1.499 ^a^	13.16 ± 2.277 ^a^	*p* > 0.2233
Non-HDL cholesterol [mg/dL]	31.29 ± 2.539	30.09 ± 2.292 ^a^	30.19 ± 3.173 ^a^	31.19 ± 2.520 ^a^	31.15 ± 2.571 ^a^	*p* > 0.8154
Triglycerides [mg/dL]	148.1 ± 39.50	134.8 ± 44.48 ^a^	116.6 ± 16.18 ^a^	114.5 ± 36.96 ^a^	138.5 ± 36.72 ^a^	*p* > 0.2434
Renal function biomarkers (1/3 affected)						
Creatinine [mg/dL]	0.373 ± 0.071	0.339 ± 0.034 ^a^	0.333 ± 0.066 ^a^	0.367 ± 0.044 ^a^	0.355 ± 0.041 ^a^	*p* > 0.5801
Urea [mg/dL]	16.12 ± 4.127	18.30 ± 3.349 ^a^	16.41 ± 2.719 ^a^	17.58 ± 3.045 ^a^	16.64 ± 2.868 ^a^	*p* > 0.6010
Uric acid [mg/dL]	0.640 ± 0.155	0.601 ± 0.202 ^a^	0.497 ± 0.113 ^a^	0.541 ± 0.121 ^a^	0.472 ± 0.069 ^a^*	* *p* > 0.0406
Pancreatic enzymes (2/2 affected)						
Amylase [U/L]	2672 ± 192.0	2441 ± 166.0 ^a^*	2341 ± 216.5 ^a^*	2363 ± 150.8 ^a^*	2395 ± 138.7 ^a^*	* *p* ≤ 0.0154
Lipase [U/L]	7.566 ± 0.537	7.956 ± 0.629 ^a^	7.486 ± 0.305 ^a^	6.855 ± 0.737 ^b^*	6.585 ± 0.320 ^b^*	* *p* ≤ 0.0209
Cardiac and muscle injury markers (0/4 affected)						
Troponin T [ng/L]	17.63 ± 5.432	16.50 ± 6.019 ^a^	16.70 ± 6.412 ^a^	19.95 ± 14.89 ^a^	20.26 ± 8.641 ^a^	*p* > 0.9605
CK-MB [U/L]	343.5 ± 98.05	381.9 ± 181.3 ^a^	330.7 ± 144.2 ^a^	408.1 ± 143.5 ^a^	340.6 ± 175.1 ^a^	*p* > 0.7536
Creatine kinase (CK) [U/L]	185.2 ± 49.97	220.0 ± 108.4 ^a^	189.0 ± 80.96 ^a^	232.2 ± 80.15 ^a^	192.3 ± 99.25 ^a^	*p* > 0.5363
Lactate dehydrogenase (LDH) [U/L]	282.6 ± 105.5	385.9 ± 215.8 ^a^	309.0 ± 160.8 ^a^	405.3 ± 150.2 ^a^	323.4 ± 198.0 ^a^	*p* > 0.2874
Electrolyte balance (3/3 affected)						
Sodium [mmol/L]	141.5 ± 0.8834	135.0 ± 3.119 ^a^*	133.8 ± 4.532 ^a^*	135.4 ± 2.192 ^a^*	134.3 ± 3.380 ^a^*	* *p* < 0.0001
Potassium [mmol/L]	4.048 ± 0.1546	4.100 ± 0.2053 ^a^	4.106 ± 0.2150 ^a^	4.259 ± 0.1956 ^b^*	4.314 ± 0.2154 ^b*^	* *p* ≤ 0.0460
Chlorides [mmol/L]	101.3 ± 1.768	96.69 ± 2.182 ^a^*	96.73 ± 3.641 ^a^*	98.11 ± 1.989 ^a^*	97.48 ± 2.303 ^a^*	* *p* ≤ 0.0189
Mineral metabolism biomarkers (1/3 affected)						
Calcium [mg/dL]	9.849 ± 0.1351	9.256 ± 0.2511 ^a^*	9.164 ± 0.3671 ^a^*	9.273 ± 0.2900 ^a^*	9.397 ± 0.4407 ^a^*	* *p* ≤ 0.0117
Inorganic phosphorus [mg/dL]	4.826 ± 0.7819	4.928 ± 0.5721 ^a^	5.052 ± 0.5043 ^a^	5.344 ± 0.5106 ^a^	5.140 ± 0.5039 ^a^	*p* > 0.1042
Magnesium [mg/dL]	2.238 ± 0.1801	2.151 ± 0.1800 ^a^	2.164 ± 0.2273 ^a^	2.340 ± 0.1961 ^a^	2.228 ± 0.1953 ^a^	*p* > 0.7851
Inflammatory and immunological biomarkers (1/2 affected)						
C-reactive protein (CRP) [mg/L]	0.216 ± 0.022	0.151 ± 0.010 ^a^*	0.151 ± 0.022 ^a^*	0.149 ± 0.019 ^a^*	0.169 ± 0.034 ^a^*	* *p* ≤ 0.0006
Rheumatoid factor (RF) [IU/mL]	6.982 ± 0.543 ^a^	6.948 ±0.896 ^a^	6.324 ± 1.093 ^a^	7.008 ± 0.455 ^a^	7.583 ± 0.863 ^a^	*p* > 0.1689
Nutritional metabolism (1/1 affected)						
Iron [µg/dL]	207.2 ± 20.21	179.1 ± 17.28 ^a^*	165.9 ± 19.98 ^a^*	173.9 ± 15.10 ^a^*	175.8 ± 20.62 ^a^*	* *p* ≤ 0.0040

Data are means ± SD, n = 10. * *p* ≤ 0.05 vs. control. Different superscript letters indicate significant differences among groups, whereas identical letters indicate no significant differences (two-way ANOVA with Tukey’s multiple comparisons test, see Supplementary Materials Table S3). LMPR vs. K (one-way ANOVA with Dunnett’s multiple-comparisons test). Blood serum samples were examined using biochemical analyses (BA400 BioSystems, Barcelona, Spain) and immunochemical assays (Cobas e411, Roche, Basel, Switzerland).

**Table 8:** Changes in body weight gain (Δ), lean mass (Δ), and fat mass (Δ) measured by NMR after supplementation in rats (mixed-effects model).

Group	Δ Body Weight (g, Mean ± SD)	* *p* vs. K	Δ NMR Lean (Mean ± SD)	* *p* vs. K	Δ NMR Fat (Mean ± SD)	* *p* vs. K
Control (K)	80.8 ± 13.1 ^a^	–	47.18 ± 8.66 ^a^	–	15.93 ± 4.79 ^a^	–
*Boldor* (LM)	90.7 ± 10.1 ^b^	0.020	50.66 ± 16.67 ^a^	0.608	19.96 ± 9.85 ^a^	0.181
*Wodan* (PR)	85.8 ± 10.8 ^a^	0.244	49.07 ± 21.37 ^a^	0.783	17.11 ± 6.41 ^a^	0.698

Data are presented as mean ± SD. * *p* ≤ 0.05. Different superscript letters indicate significant differences among groups, whereas identical letters indicate no significant differences (one-way ANOVA with Dunnett’s multiple-comparisons test). Changes in body weight, fat mass, and lean mass (Δ) were calculated as the difference between post-supplementation and baseline values. Statistical analysis was performed using a linear mixed-effects model, with time (pre- vs. post-supplementation) as a within-subject factor and experimental group as a fixed factor. The pattern of body weight gain (Δ), lean mass (Δ), and fat mass (Δ) followed the order: *Boldor* > *Wodan* > *control*.

**Table 9:** Liver, heart, spleen and kidney weights.

Group	Liver (Mean ± SD)	* *p*	Heart (Mean ± SD)	* *p*	Spleen (Mean ± SD)	* *p*	Kidney (Mean ± SD)	* *p*
Control (K)	10.75 ± 1.29 ^a^	–	1.25 ± 0.20 ^a^	–	0.692 ± 0.100 ^a^	–	2.02 ± 0.16 ^a^	–
*Boldor* (LM)	11.01 ± 1.09 ^a^	0.569	1.31 ± 0.16 ^a^	0.405	0.726 ± 0.074 ^a^	0.296	2.18 ± 0.19 ^b^	0.028
*Wodan* (PR)	10.64 ± 0.76 ^a^	0.775	1.31 ± 0.20 ^a^	0.456	0.767 ± 0.077 ^b^	0.033	2.14 ± 0.15 ^b^	0.043

Data are presented as mean ± SD. * *p* ≤ 0.05. Different superscript letters indicate significant differences among groups, whereas identical letters indicate no significant differences (one-way ANOVA with Dunnett’s multiple-comparisons test). Kidney weight was higher in the *Boldor* (*p* = 0.028) and *Wodan* (*p* = 0.043) groups, while spleen weight was higher in the *Wodan* group (*p* = 0.033). No significant differences were observed for liver or heart weight. Mean value trends were liver (*Boldor* > Control > *Wodan*), heart (*Wodan* > *Boldor* > *Control*), spleen (*Wodan* > *Boldor* > *Control*), and kidney (*Boldor* > *Wodan* > *Control*).

**Table 10:** Feed efficiency ratio in experimental groups.

Group	Weight Gain (g)	Feed Intake (g/day)	Total Feed (g)	FER (Mean ± SD)
Control (K)	80.8 ± 13.1 ^a^	17.33 ± 1.11 ^a^	788 ^a^	0.102 ± 0.012 ^a^
*Boldor* (LM)	90.7 ± 10.1 ^b^	17.83 ± 0.65 ^a^	811 ^a^	0.112 ± 0.011 ^a^
*Wodan* (PR)	85.8 ± 10.8 ^a^	17.64 ± 0.59 ^a^	803 ^a^	0.107 ± 0.013 ^a^

Data are presented as mean ± SD. *p* > 0.05. Different superscript letters indicate significant differences among groups, whereas identical letters indicate no significant differences (one-way ANOVA with Dunnett’s multiple-comparisons test). Average daily feed intake was calculated as total feed consumption divided by the number of feeding days. Feed efficiency ratio (FER) was calculated as body weight gain divided by total feed intake during the experimental period. The calculated FER was approximately 0.11, indicating that 0.11 g of body weight gain was obtained per gram of feed consumed. Trend in feed efficiency ratio (FER): *Boldor* > *Wodan* > control. The *Boldor* group demonstrated the highest feed efficiency ratio, even though its feed intake did not exceed that of the control group.

**Table 11:** Pharmacological parameters obtained from nonlinear regression analysis of concentration–response curves in control (K) and treatment groups (*Boldor* and *Wodan*).

Group	Emax	−logEC50	AUC
Control (K)	55.85 ± 3.160 ^a^	7.679 ± 0.1594 ^a^	150.3 ± 20.59 ^a^
*Boldor* (LM)	60.39 ± 3.129 ^a^	7.813 ± 0.1463 ^a^	171.6 ± 22.39 ^a^
*Wodan* (PR)	58.19 ± 2.952 ^a^	7.785 ± 0.1425 ^a^	164.1 ± 16.97 ^a^

Data are presented as mean ± SEM. Concentration–response curves were analyzed using nonlinear regression with a four-parameter logistic model (4PL). Emax represents the maximal response, pEC50 (−logEC50) is the negative logarithm of the half-maximal effective concentration, and EC50 is the concentration producing 50% of the maximal response (nM). AUC denotes the area under the concentration–response curve calculated across the tested concentration range. *p* > 0.05. Different superscript letters indicate significant differences among groups, whereas identical letters indicate no significant differences (one-way ANOVA with Dunnett’s multiple-comparisons test).

**Table 12:** The isolated perfused heart.

Group	Heart Rate (HR) (bpm)	LVDP (mmHg)	+dP/dtmax (mmHg/s)	LVEDP (mmHg)	Coronary Flow (ml/min)	RPP
Control (K)	280 ± 12 ^a^	130 ± 5 ^a^	4400 ± 252 ^a^	6–8 ^a^	12–15 ^a^	37,600 ± 2100 ^a^
*Boldor* (LM)	278 ± 11 ^a^	125 ± 4 ^a^	4200 ± 248 ^a^	6–8 ^a^	13–15 ^a^	35,900 ± 1600 ^a^
*Wodan* (PR)	285 ± 13 ^a^	126 ± 5 ^a^	4350 ± 240 ^a^	6–8 ^a^	14–15 ^a^	34,500 ± 1700 ^a^

Values are expressed as mean ± SEM. *p* > 0.05. Different superscript letters indicate significant differences among groups, whereas identical letters indicate no significant differences (one-way ANOVA with Dunnett’s multiple-comparisons test).

## Data Availability

The data presented in this study are available in this article and its Supplementary Materials.

## Supplementary Materials

The following supporting information can be downloaded at: https://www.mdpi.com/article/10.3390/nu18122016/s1, Figure S1: Blood serum biomarkers of lipid metabolism and cardiac (A–E) and skeletal muscle injury (F–I). Data were analyzed using one-way ANOVA with Tukey’s multiple-comparisons test. No statistically significant differences were detected between groups; Table S1: Dietary composition (%); Table S2: Concentrations of selected trace elements (Fe, Zn, Cu, Cr, Ni, Se) and potentially toxic elements (Pb, As, Cd, Sb) in experimental diets across treatment groups subjected to foliar application of the selenium-based plant growth stimulator; Table S3. Two-way ANOVA analysis.

## Abbreviations

The following abbreviations are used in this manuscript:

ACh	Acetylcholine
ANOVA	Analysis of Variance
As	Arsenic
AUC	Area Under the Curve
BBCH	Federal Biological Research Centre
Cd	Cadmium
CCRCS	Cumulative Concentration–Response Curves
Cr	Chromium
Cu	Copper
EC50	Half–maximal Effective Concentration
Emax	Maximal Response
Fe	Iron
FER	Feed Efficiency Ratio
HR	Heart Rate
ICP–MS/ORS	Inductively Coupled Plasma Mass Spectrometry with Octopole Reaction System
IPH	Isolated Perfused Heart
K	Control Group
KCl	Potassium Chloride
KHS	Krebs–Henseleit Solution
LVDP	Left Ventricular Developed Pressure
LVEDP	Left Ventricular End–Diastolic Pressure
NA	Noradrenaline
Ni	Nickel
NMR	Nuclear Magnetic Resonance
NO	Nitric Oxide
ORS	Octopole Reaction System
Pb	Lead
RPP	Rate–Pressure Product
Sb	Antimony
SD	Standard Deviation
SEM	Standard Error of the Mean
Se	Selenium
Zn	Zinc
WKY	Wistar–Kyoto Rats
