# Supplementary material for: Preliminary Assessment of Red Beetroot Supplementation and Cultivar Effects in Low-Protein-Fed WKY Rats
Source: Nutrients. 2026 Jun 21;18(12):2016. doi: 10.3390/nu18122016 (PMC13304773; doi:10.3390/nu18122016)
Supplement: Supplementary file 1 [file nutrients-18-02016-s001.zip › Figure S1.pdf]

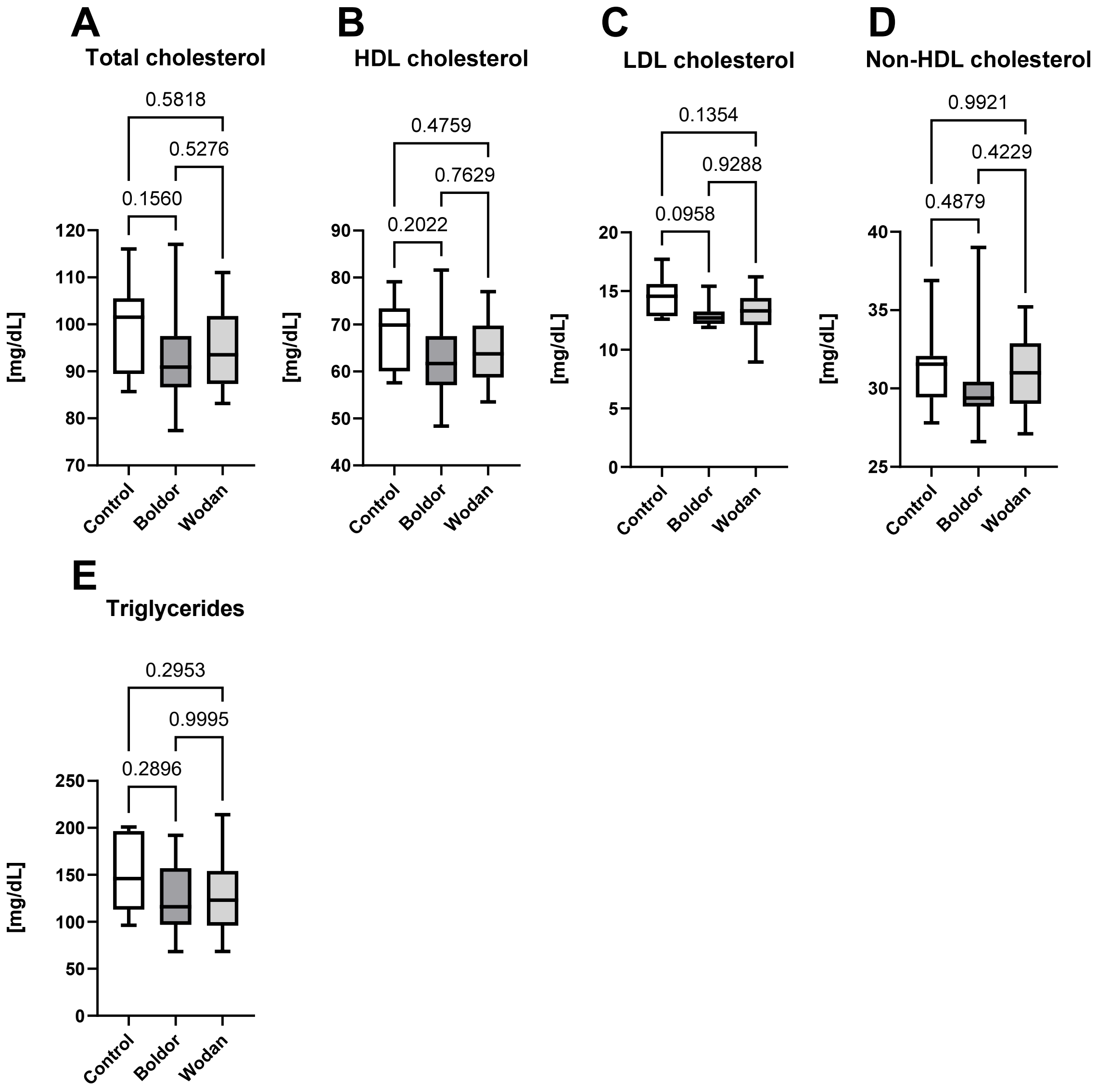

A
Total cholesterol
0.5818
0.5276
0.1560
[mg/dL]
120
110
100
90
80
70
Control
Boldor
Wodan
B
HDL cholesterol
0.4759
0.7629
0.2022
[mg/dL]
90
80
70
60
50
40
Control
Boldor
Wodan
C
LDL cholesterol
0.1354
0.9288
0.0958
[mg/dL]
20
15
10
5
0
Control
Boldor
Wodan
D
Non-HDL cholesterol
0.9921
0.4229
0.4879
[mg/dL]
40
35
30
25
Control
Boldor
Wodan
E
Triglycerides
0.2953
0.9995
0.2896
[mg/dL]
250
200
150
100
50
0
Control
Boldor
Wodan

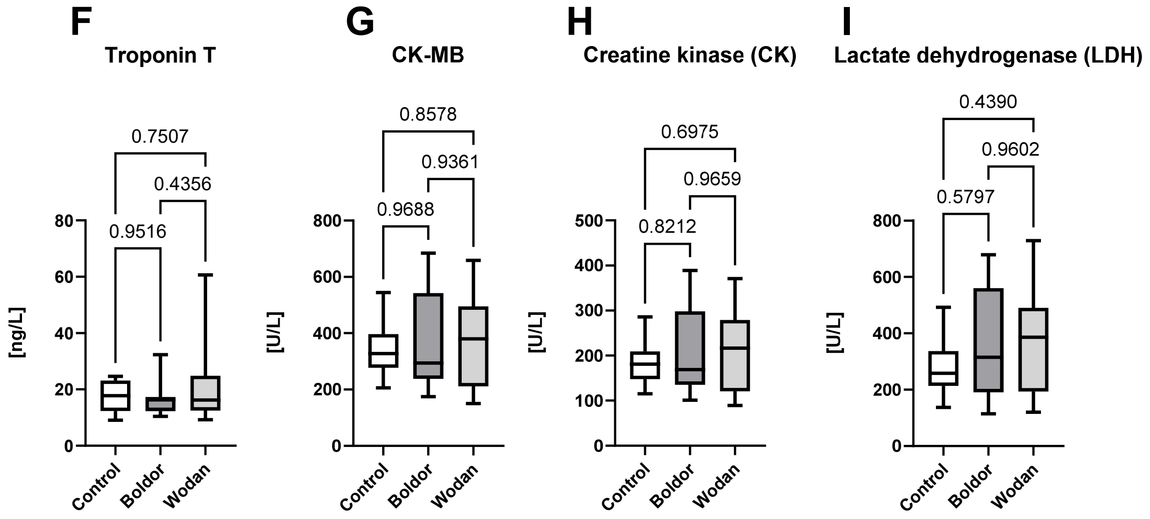

Figure S1. Blood serum biomarkers of lipid metabolism and cardiac (A-E) and skeletal muscle injury (F-I). Data were analyzed using one-way ANOVA with Tukey's multiple-comparisons test. No statistically significant differences were detected between groups.
