# Supplementary material for: Preliminary Assessment of Red Beetroot Supplementation and Cultivar Effects in Low-Protein-Fed WKY Rats
Source: Nutrients. 2026 Jun 21;18(12):2016. doi: 10.3390/nu18122016 (PMC13304773; doi:10.3390/nu18122016)
Supplement: Supplementary file 1 [file nutrients-18-02016-s001.zip › Table S2cc.pdf]

Table S2. Concentrations of selected trace elements (Fe, Zn, Cu, Cr, Ni, Se) and potentially toxic elements (Pb, As, Cd, Sb) in experimental diets across treatment groups subjected to foliar application of the selenium–based plant growth stimulator.

Assay	Control (K)	Boldor 1 (L)	Boldor 3 (M)	Wodan 1 (P)	Wodan 3 (R)	Cultivar effect	Se effect	Interaction Cultivar x Se	*LMPR vs K			
						two-way ANOVA			one-way ANOVA			
									L	M	P	R
Fe	72.86	75.98^{a}	75.05^{a}	128.7^{b}	131.3^{b}	$p < 0.0001$	$p = 0.661$	$p = 0.368$			<0.0001	<0.0001
	2.448	2.886	7.960	2.842	3.460							
Zn	34.76	35.08^{a}	36.09^{a}	37.47^{b}	38.56^{b}	$p = 0.003$	$p = 0.149$	$p = 0.983$			0.0357	0.0026
	0.9987	1.446	3.136	0.9063	1.430							
Cu	5.807	5.757^{a}	5.990^{a}	6.612^{b}	7.299^{b}	$p < 0.000001$	$p = 0.175$	$p = 0.164$			0.0426	<0.0001
	0.1590	0.2425	0.5289	0.1021	0.2502							
Cr	0.3987	0.8213^{b}	0.8230^{b}	2.046^{c}	1.621^{c}	$p < 0.0001$	$p = 0.289$	$p = 0.404$	<0.0001	<0.0001	<0.0001	<0.0001
	0.02277	0.02715	0.08933	0.04457	0.04158							
Ni	0.6284	0.5487^{a}	0.6508^{a}	0.6439^{a}	0.5159^{a}	$p = 0.72$	$p = 0.75$	$p = 0.28$				
	0.04375	0.04740	0.1119	0.03411	0.02438							
Se	0.08834	0.1226^{a}	0.09807^{a}	0.1160^{a}	0.08120^{a}	$p = 0.491$	$p = 0.191$	$p = 0.476$				
	0.01641	0.03203	0.03040	0.04123	0.02250							
Pb	0.01136	0.04883^{b}	0.05781^{b}	0.08771^{c}	0.09455^{c}	$p < 0.0001$	$P = 0.335$	$p = 0.34$	<0.0001	<0.0001	<0.0001	<0.0001
	0.004054	0.006153	0.009421	0.008063	0.009275							
As	0.008896	0.02201^{b}	0.02582^{b}	0.06464^{c}	0.07128^{c}	$p < 0.0001$	$p = 0.274$	$p = 0.485$	0.0021	0.0001	0.0001	0.0001
	0.002867	0.003716	0.005806	0.006618	0.008131							
Cd	0.003684	0.006823^{a}	0.005678^{a}	0.01433^{b}	0.01430^{b}	$p < 0.0001$	$p = 0.112$	$p = 0.586$			<0.0001	<0.0001
	0.001465	0.003279	0.001481	0.003163	0.002463							
Sb	0.002101	0.002487^{a}	0.004475^{a}	0.005475^{b}	0.005140^{b}	$p = 0.004$	$p = 0.221$	$p = 0.117$			0.0001	0.0005
	0.001038	0.000759	0.000868	0.001277	0.001430							

Data are presented as mean ± standard deviation (SD). $^{*}p \leq 0.05$. Different superscript letters indicate significant differences among groups, whereas identical letters indicate no significant differences (two-way ANOVA with Tukey's multiple comparisons test). Control vs red beet cultivars (*Boldor* and *Wodan*) (one-way ANOVA with Dunnett's multiple comparisons test). Foliar application of the selenium–based plant growth stimulator did not significantly increase selenium or other element concentrations in beet roots.
