# Supplementary material for: Preliminary Assessment of Red Beetroot Supplementation and Cultivar Effects in Low-Protein-Fed WKY Rats
Source: Nutrients. 2026 Jun 21;18(12):2016. doi: 10.3390/nu18122016 (PMC13304773; doi:10.3390/nu18122016)
Supplement: Supplementary file 1 [file nutrients-18-02016-s001.zip › Table S1.pdf]

Table S1. Dietary composition (%).

Ingredient	Control (%)	*Wodan* and *Boldor* (%)
Casein	8.0	8.0
Methionine	0.3	0.3
Rapeseed oil	2.0	2.0
Lard	10	10
Sucrose	11	11
Cellulose	5.0	5.0
Mineral mixture	3.5	3.5
Vitamin mixture	1.0	1.0
Choline chloride	0.2	0.2
Beetroot	0	4.0
Corn starch	59	55
Total	100	100

Experimental diets were prepared from individual ingredients. Mineral mixture (%): Iron citrate [16.7% Fe] – 31.0; Zinc carbonate (ZnCO3) [56% Zn] – 4.5; Manganese carbonate (MnCO3) [44.4% Mn] – 23.4; Copper carbonate (CuCO3) [55.5% Cu] – 1.85; Potassium iodide (KI) – 0.04; Citric acid – 39.21.
