# Supplementary material for: Preliminary Assessment of Red Beetroot Supplementation and Cultivar Effects in Low-Protein-Fed WKY Rats
Source: Nutrients. 2026 Jun 21;18(12):2016. doi: 10.3390/nu18122016 (PMC13304773; doi:10.3390/nu18122016)
Supplement: Supplementary file 1 [file nutrients-18-02016-s001.zip › Table S3.pdf]

Table S3. Two-way ANOVA analysis.

**Assay**	**Cultivar effect**	**Se effect**	**Interaction**
Alanine aminotransferase (ALT) [U/L]	p = 0.12	p = 0.11	p = 0.16
Aspartate aminotransferase (AST) [U/L]	p = 0.91	p = 0.39	p = 0.29
Alkaline phosphatase (ALP) [U/L]	p = 0.34	p = 0.32	p = 0.08
Total bilirubin [mg/dL]	p = 0.08	p = 0.60	p = 0.72
Direct bilirubin [mg/dL]	p = 0.44	p = 0.56	p = 0.18
Albumin [g/dL]	p = 0.31	p = 0.57	p = 0.22
Total protein [g/dL]	p = 0.83	p = 0.74	p = 0.12
Total cholesterol [mg/dL]	p = 0.79	p = 0.94	p = 0.21
HDL cholesterol [mg/dL]	p = 0.95	p = 0.82	p = 0.14
LDL cholesterol [mg/dL]	p = 0.08	p = 0.73	p = 0.64
Non-HDL cholesterol [mg/dL]	p = 0.19	p = 0.73	p = 0.21
Triglycerides [mg/dL]	p = 0.69	p = 0.28	p = 0.42
Creatinine [mg/dL]	p = 0.59	p = 0.81	p = 0.41
Urea [mg/dL]	p = 0.82	p = 0.19	p = 0.58
Uric acid [mg/dL]	p = 0.29	p = 0.07	p = 0.29
Amylase [U/L]	p = 0.67	p = 0.55	p = 0.89
**Lipase [U/L]**	**p < 0.0001**	p = 0.67	p = 0.17
Troponin T [ng/L]	p = 0.84	p = 0.49	p = 0.63
CK-MB [U/L]	p = 0.94	p = 0.62	p = 0.63
Creatine kinase (CK) [U/L]	p = 0.99	p = 0.56	p = 0.72
Lactate dehydrogenase (LDH) [U/L]	p = 0.96	p = 0.31	p = 0.27
Sodium [mmol/L]	p = 0.28	p = 0.39	p = 0.12
**Potassium [mmol/L]**	**p = 0.03**	p = 0.14	p = 0.16
Chlorides [mmol/L]	p = 0.18	p = 0.46	p = 0.27
Calcium [mg/dL]	p = 0.72	p = 0.91	p = 0.31
Inorganic phosphorus [mg/dL]	p = 0.09	p = 0.18	p = 0.11
Magnesium [mg/dL]	p = 0.11	p = 0.26	p = 0.36
C-reactive protein (CRP) [mg/L]	p = 0.36	p = 0.08	p = 0.26
Rheumatoid factor (RF) [IU/mL]	p = 0.70	p = 0.93	p = 0.64
Iron [µg/dL]	p = 0.74	p = 0.52	p = 0.74
